# Linking Identity Leadership and Team Performance: The Role of Group-Based Pride and Leader Political Skill

**DOI:** 10.3389/fpsyg.2021.676945

**Published:** 2021-10-01

**Authors:** Liang Hou, Lynda Jiwen Song, Guoyang Zheng, Bei Lyu

**Affiliations:** ^1^School of Business, Renmin University of China, Beijing, China; ^2^Leeds University Business School, University of Leeds, Leeds, United Kingdom; ^3^School of Economics and Management, Huaibei Normal University, Huaibei, China; ^4^Business School, Henan University, Kaifeng, China; ^5^Chinese Graduate School, Panyapiwat Institute of Management, Nonthaburi, Thailand

**Keywords:** identity leadership, group-based pride, team performance, leader political skill, moderated mediation model

## Abstract

Recent trends in the leadership literature have promoted a social identity approach of leadership that views leadership as the process of representing, advancing, creating, and embedding a sense of shared identity within a group. However, a few empirical studies explore how and when global identity leadership affects team performance at the workplace. To address this lacuna, we used multi-source and two-wave data among 81 teams to explore the role of group-based pride and leader political skill in the association between identity leadership and team performance. The results suggest that identity leadership positively predicts team performance through a mediating role of group-based pride. Furthermore, leader political skill moderates the indirect effect of group-based pride such that the effect is stronger when leader political skill is high rather than low. Finally, several theoretical and practical implications of this study are discussed, and future research directions are also suggested.

## Introduction

The essence of leadership is often considered as the process of exerting influence on others (Yukl, 2012). Effective leaders use their abilities to influence the thoughts, feelings, and behaviors of followers motivating followers toward shared interests or collective goals (Chemers, 2001). Growing research suggests that altering the identity and self-concept of followers is a key determinant of leadership to exert influence (e.g., van Knippenberg and Hogg, 2003; Epitropaki et al., 2017). One's subjective self-concept can come from one's personal identity (i.e., a unique individual as “I” and “me”) as well as from one's social identity (i.e., group membership as “we” and “us”; Turner, 1982). Leader position is more likely to be considered as a center on the construction and enactment of a shared social identity for group members, which, in turn, promotes group efforts and group-oriented behaviors (Ellemers et al., 2004; Haslam et al., 2020). In line with this claim, researchers propose a social identity approach of leadership that views leadership as the process of representing, advancing, creating, and embedding a sense of shared identity within a group (Hogg, 2001; van Dick and Kerschreiter, 2016; Haslam et al., 2020). More specifically, Steffens et al. (2014) introduced this type of leadership as identity leadership and specified four interrelated dimensions: *identity prototypicality, identity advancement, identity impresarioship*, and *identity entrepreneurship*.

Although a social identity approach of leadership has attracted considerable attention from researchers in the past two decades, most previous studies focus on theoretical articulation and the single dimension of leader identity prototypicality (Steffens et al., 2014). In this regard, existing empirical evidence has suggested that leader identity prototypicality contributes to leadership effectiveness (van Knippenberg, 2011; Steffens et al., 2021). However, other equally significant aspects of identity leadership are neglected (Steffens et al., 2014). Leaders not only need to present a group prototypicality but also need to create, develop, and embed a shared group identity to achieve influence. Recent research argues that identity leadership, as a multidimensional construct, goes beyond leader prototypicality and has developed a reliable instrument using cross-cultural samples (Steffens et al., 2014; van Dick et al., 2018), which provides a comprehensive theoretical and conceptual basis for further research. Unfortunately, there are still a few studies to explore the underlying processes that global identity leadership as a higher-order construct affects team effectiveness. This shortage of empirical research on global identity leadership pushes us to explore how and when identity leadership affects team performance.

Traditionally, leadership theory predominantly focuses on the characteristics of leaders, such as their personalities, styles, and behaviors. The social identity approach of leadership goes beyond the traditional leadership models and instead focuses on the capacity of leaders to influence followers (Steffens et al., 2020), which reflects the essence of leadership (Bennis, 2003). Through the test of global identity leadership, we can advance theoretical development on the social identity approach of leadership and provide a possible pathway to translate this approach into practice for leadership training or intervention (Haslam et al., 2017). Specifically, our research provides three theoretical contributions to the related literature. Firstly, this study examines the influence of identity leadership on team performance in work teams. Initial research on identity leadership has examined its impact on employee attitudes and behaviors, such as identification with team, job satisfaction, organizational citizenship behavior, and innovation (Steffens et al., 2014; van Dick et al., 2018). More recently, several studies use athlete teams to explore the influence of identity leadership on team function and effectiveness (Fransen et al., 2020; Miller et al., 2020). It is uncertain whether these findings can be generalized to the work teams. The current study helps to reveal the benefits of identity leadership on team performance in workplace settings.

Secondly, the social identity approach provides an appropriate theoretical framework to explain the influence mechanism of identity leadership. Similar to identity leadership, group-based emotion is also a construct rooted in the social identity approach (Campo et al., 2019; Smith and Mackie, 2020). Group-based emotions are dependent on one's self-categorization as a group member and emerge in response to situations or events that perceived relevance for one's group (Mackie et al., 2000). Although past research has found the influence of leadership on general group emotions (e.g., group affective tone; Collins et al., 2013), group-based emotions have been overlooked. As suggested by Kuppens and Yzerbyt (2014), group-based emotions and general group emotions are significantly different in terms of concept and measurement. In the current study, we use the framework of the social identity approach, examining a mediating role of the group-based emotion (i.e., group-based pride) in the relationship between identity leadership and team performance.

Finally, we examine the moderating effect of leader political skill in the relationship between identity leadership and group-based pride. Although, existing studies suggest the influence of identity leadership on team effectiveness, the impacts may depend on other boundary conditions. For example, team characteristics, followers' characteristics, and situational factors are often considered to be significant moderators in the link between leadership behavior and team effectiveness (e.g., Schaubroeck et al., 2007; Pratoom, 2018). However, the characteristic of leaders is seldom considered. Political skill may be one of the most critical capabilities for leaders to exert influence on others (Bing et al., 2011). Thus, we select the leader's political skill as a significant boundary condition because it helps us to explain why some leaders can choose appropriate behaviors and use them more effectively. These findings contribute to clarifying an interactive effect of leadership behavior and the characteristics of leaders in predicting team performance.

## Theory and Hypotheses

### The Social Identity Approach

The social identity approach offers a theoretical framework that integrates several compatible and interrelated theories, such as self-categorization theory (Turner et al., 1987), social identity theory (Tajfel and Turner, 1986), and intergroup relations theory (Mackie et al., 2000). The social identity approach mainly outlines how group membership affects one's self-concept (Hogg, 2001). Building on the core insight of this approach, a social identity model of leadership argues that leadership involves the process of using the capacities of leaders to represent, advance, create, and embed a sense of shared social identity within a group (Hogg, 2001; van Knippenberg and Hogg, 2003; Haslam et al., 2020).

Furthermore, social identity is an important part of the self-concept, which stems from “an individual's knowledge that belongs to certain groups together with emotional and value significance to him of this group membership” (Tajfel, 1982, p. 292). On one hand, social identification, as a cognitive process of social identity, involves the extent to that one uses a group membership to define oneself (Tajfel and Turner, 1986). On the other hand, salient group membership provides a basis for team members to experience group-based emotions (Mackie et al., 2000). Specifically, when one belongs to and is identified with a certain group, the appraisal of group-relevant events or characteristics will elicit one's group-based emotions. These group-based emotions are often shared among group members, which in turn motivate group-relevant behaviors toward ingroup or outgroup (Mackie et al., 2000). For example, group-based pride predicts in-group favoring intentions (Harth et al., 2013).

### Identity Leadership and Group-Based Pride

Identity leadership exerts an influence on followers effectively primarily by making them think, feel, and behave as group members (i.e., their shared social identity as “we” and “us”) rather than unique individuals (i.e., their personal identity as “I” and “me”) (Haslam et al., 2020). More specifically, the leader shares social identity within a group through four aspects: (a) *representing ingroup prototypicality* (i.e., representing unique qualities that define the group and group membership); (b) *acting as ingroup champions* (i.e., advancing and promoting shared interests of the group); (c) *crafting a sense of shared identity* (i.e., bringing group members together by creating a shared sense of “we” and “us”); and (d) *embedding shared identity* (i.e., establishing structures, events, and activities that value the existence of the group and allow group members to live out their membership) (Steffens et al., 2014). While a leader engages in identity leadership behaviors, followers will gain a sense of shared identity by considering themselves as a member of the group and highlighting the unique, special, and distinctive characteristics of one's group (van Dick et al., 2018). According to the social identity approach, on one hand, individuals use their group membership to define themselves and determine their self-worth (Turner, 1982). A shared sense of “we” and “us” deriving from identity leadership will enable group members to view the group's achievements and status as like themselves. On the other hand, to maintain or achieve a positive self-concept, group members are motivated to have favorable comparisons between their ingroup and some relevant outgroups (Tajfel et al., 1979; Abrams and Hogg, 1988). Specifically, individuals tend to positively evaluate their ingroup, make their ingroup positively distinctive from outgroups, and tend to leave their existing group or engage in social actions to make desirable changes if social identity is unsatisfactory (Tajfel et al., 1979). As such, engaging in identity leadership can help group members to form a shared sense of “we” and “us” and develop the identity of a positive group.

Group-based pride is rooted in one's group membership (e.g., Leary et al., 2014) and generated from acknowledging the positive aspects of the identity of a group (e.g., group accomplishments, group value, or group status) (Cehajić-Clancy et al., 2011). Group-based pride reflects the positive evaluation of individuals to the status or achievement of their group. Existing studies have suggested that group-based pride originates from the consideration of the ingroup as moral (Leach et al., 2007), as having a legitimate advantage over outgroups (Harth et al., 2008), or as succeeding in a match (Bravo et al., 2020). As discussed earlier, we anticipate that when a team leader engages in identity leadership, team members will internalize their group membership and view their ingroup as a valued and positive social group, in turn, elicits group-based pride. More specifically, we expect that:

*Hypothesis 1: Identity leadership is positively related to group-based pride*.

### Group-Based Pride and Team Performance

A fundamental assumption of most emotion-related theories is that emotions are functional (Keltner and Haidt, 1999). Different emotions may promote distinct types of adaptive behaviors and social outcomes. Concretely, pride is usually considered as a rank-related emotion (Oveis et al., 2010), which drives individuals to participate in behaviors that are aimed at maintaining a positive image and attaining a higher status (Anderson et al., 2015). Accordingly, group-based emotions find their functions in regulating the attitudes and behaviors of group members toward ingroup and outgroup (Smith and Mackie, 2020). Group-based pride is dependent upon the group membership of an individual and occurs in response to situations that perceived high achievements and the status of the group (Mackie et al., 2000; Harth et al., 2013).

We expect that employees' pride in their group will promote team performance in the following two ways. Firstly, group-based pride offers necessary motivation for driving a group effort on team tasks and engenders perseverance for future success (Williams and DeSteno, 2008). Individuals with a high level of pride in their group usually perceive the group as important, meaningful, effective, and worthwhile. To sustain and further strengthen the status of one's group, team members are motivated to make arduous efforts on their work and engage in activities that help the group to meet its objectives. Indeed, previous studies have suggested that the feeling of pride stimulates task participation and task efforts (e.g., Anderson et al., 2015). Moreover, group-based pride serves as an incentive to pursue further success despite short-term costs (Williams and DeSteno, 2008). As Fredrickson (2001) suggested, pride might spur team members to dream of further achievement and lead to greater perseverance for future success. As such, group-based pride offers necessary motivation for team efforts and perseverance, which not only has a positive impact on the current team performance but also provides an important basis for achieving future team performance.

Secondly, group-based pride promotes individuals to prefer their ingroup members, which increases group cooperation and group cohesion. Prior studies suggest that group-based pride enhances ingroup favoritism (Harth et al., 2008, 2013). For instance, people tend to evaluate ingroup members more positively, give more support and resources to ingroup members, and are more willing to cooperate with ingroup members (Balliet et al., 2014). Indeed, in comparison to the joy or enjoyment condition, prior research finds that pride condition improves the perceived importance of cooperation, and, as a result, promotes cooperative choices in a social dilemma (Dorfman et al., 2014). Group-based pride is an important effective function of cohesion as well (Severt and Estrada, 2015). More importantly, previous studies have suggested that group cooperation and cohesion are indispensable to work team performance and success (Mathieu et al., 2015; Lin et al., 2017). We, therefore, hypothesize that group-based pride positively contributes to team performance.

*Hypothesis 2: Group-based pride is positively related to team performance*.

Taken together, we expect that group-based pride mediates the relationship between identity leadership and team performance. As suggested by Cehajić-Clancy et al. (2011), group-based emotions can play a mediating role between self-categorization factors and action tendency. Thus, we propose that:

*Hypothesis 3: Group-based pride mediates the relationship between identity leadership and team performance*.

### The Moderating Effect of Leader Political Skill

Although the expectation of identity leadership may promote a sense of shared identity and elicit group-based pride, political skill is equally important for leaders to successfully influence the emotions and behaviors of team members. Specifically, we propose that leader political skill as a boundary condition moderates the association between identity leadership and group-based pride. Political skill is described as “the ability to effectively understand others at work, and to use such knowledge to influence others to act in ways that advance personal and/or organizational objectives” (Ferris et al., 2005, p.127). Existing studies have shown that political skill plays a moderating role in the effectiveness of leadership and social influence tactics (e.g., Buch et al., 2016). For example, political skill mitigates the negative effects of transactional leader–member exchange (LMX) (Buch et al., 2016), makes proactive employees more effective (Sun and van Emmerik, 2015), and strengthens a recipient's perception of ingratiatory strategies (Treadway et al., 2007).

As a social ability, political skill enhances the achievement of the goals of an individual and/or organization through their understanding and influence of others in social interactions. Leaders engaging in identity leadership behaviors inspire the pride of followers through delivering a sense of shared identity within a group, which probably depends on the leader political skill. Political skill includes four critical dimensions: *social astuteness, interpersonal influence, networking ability*, and *apparent sincerity* (Ferris et al., 2005). All four dimensions are important in the process of leaders exerting influence on the social identity of followers. Specifically, we contend that a leader's political skill can strengthen the association between identity leadership and group-based pride through two aspects: access to information and effective delivery (Sun and van Emmerik, 2015). Firstly, politically skilled leaders can use their social astuteness to accurately understand team members and their social situations, and utilize their networking ability to access various information and accurately recognize the needs of their group and organization (Ferris et al., 2005). As such, leaders with high social astuteness and networking ability can use sufficient information to exactly identify the unique qualities, values, and core goals of the group, clearly define the contents and boundaries of the group identity, and provide an appropriate reality for the group identity. Given that the salience of group identity is a basic condition of group-based pride, leader political skill is expected to strengthen the relationship between identity leadership and group-based pride.

Secondly, interpersonal influence and apparent sincerity allow leaders to adapt their behaviors and make them be perceived as genuine and trustworthy to subtly influence others (Munyon et al., 2015). Politically skilled leaders not only exactly know what should do in different situations to present and craft group membership, but also exactly know how to do it to advance and embed a sense of shared identity. Political skill is especially useful in situations requiring interpersonal influence (Bing et al., 2011). Benefiting from interpersonal influence and apparent sincerity, politically skilled leaders can deliver their ideas and actions to their followers more effectively. Therefore, identity leaders who have a high-level political skill will use interpersonal influence tactics and show sincerity and credibility when constructing a social identity within the group, fostering followers to more identify with their group and generate higher group-based pride. Overall, we propose that:

*Hypothesis 4: Leader political skill moderates the positive relationship between identity leadership and group-based pride such that the relationship is stronger when leader political skill is high rather than low*.

**Figure 1:**
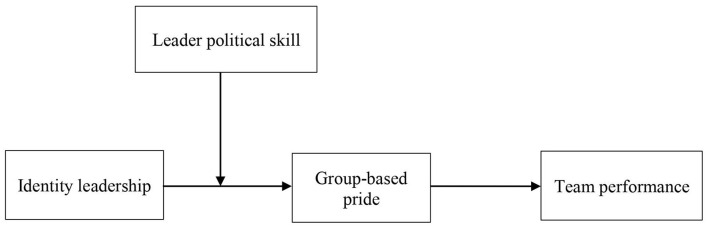
Proposed theoretical model.

Taken together, the current study proposes an integrated moderated mediation model in which group-based pride mediates the influence of identity leadership on team performance, whereas leader political skill moderates the mediation. In other words, the indirect effect of identity leadership on team performance *via* group-based pride will be more strongly when the leader's political skill is high rather than low. Therefore, we propose Hypothesis 5. Figure 1 summarizes our theoretical model.

*Hypothesis 5: Leader political skill moderates the indirect effect of identity leadership on team performance via group-based pride such that the indirect effect is stronger when leader political skill is high rather than low*.

## Methods

### Participants and Procedures

Our sample was composed of work teams from several companies involved in the law firms and legal departments. A work team refers to a direct supervisor and two or more team members who share common goals, perform interdependent tasks, and are responsible for collective outcomes (Kozlowski and Bell, 2003). We received a strong support from all participants as well as the human resources departments of companies during the process of data collection. To minimize common method bias effects, we prepared and conducted two different sets of questionnaires to supervisors and their team members (Antonakis et al., 2010). Each questionnaire was assigned with a numeric identification code, which could be used for matching by the research team, such that participants would remain confidential in the research. We collected two-wave data at different times. At Time 1, team members reported their demographic information, perceived the identity leadership of their supervisors, and group-based pride. At the same time, team supervisors assessed their demographic information and team performance. At Time 2, 1 month later, team supervisors reported their own political skill. In the questionnaire of supervisors, we separated Time 1 and Time 2 surveys to control the length of each survey and increase response rates. Leader political skill was assessed at Time 2 because it is relatively stable and unlikely to change in a short-term time (Liu et al., 2007).

We initially distributed 84 questionnaires to team supervisors and 314 questionnaires to team members, and then received 83 responses from supervisors and 310 responses from team members. In the current study, we included those work teams that meet two criteria: (1) the direct supervisor of the team completed the measures gauged in the supervisor survey and (2) at least two team members completed the variables assessed in the employee survey. In addition, we used attention check questions in the supervisor and team member surveys to eliminate those who were not serious. Finally, 81 effective teams were included in the study (i.e., 81 supervisors and 292 team members; their effective rate is 96.4 and 93.0%, respectively). The averaged team size of the final sample was 3.59 (SD = 1.23) ranging from 2 to 9 members. In the employee sample, 43.1% of the team members were male; the average age of the members was 28.18 years (SD = 3.83); and average job tenure in the team was 2.22 years (SD = 1.59). In the supervisor sample, 61.3% of the supervisors were male, the average age of the supervisors was 38.04 years (SD = 5.97), and the average job tenure in the team was 5.65 years (SD = 3.65).

### Measures

We employed a standard translation-back-translation procedure (Brislin, 1970) to translate all measures into Chinese because they were initially written in English. All participants were asked to assess items using a six-point Likert-type scale (from 1 = strongly disagree to 6 = strongly agree).

### Identity Leadership

We used a short four-item scale taken from Steffens et al. (2014) to assess identity leadership. Team members were asked to evaluate the identity leadership behaviors of their supervisor. A sample item was “This leader is a model member of our group.” The short scale has been used with high reliability and calculated the total score to represent global identity leadership in previous research (Fransen et al., 2020). Our result suggested that the Cronbach coefficient of global identity leadership was 0.78. Moreover, we conducted confirmatory factor analyses (CFA) to examine the structural validity of this scale. The result suggested that the four-item identity leadership scale had a good fit index [${\text{χ}}_{\left(2\right)}^{2}$ = 12.95, CFI = 0.97, TLI = 0.91, and SRMR = 0.04].

### Group-Based Pride

Group-based pride was assessed with a four-item scale developed by Tyler et al. (1996). This scale is used with high reliability in past research (Steven et al., 2009). We asked team members to evaluate the extent to that they agree with each item about their workgroup. A sample item was “I feel proud to be a part of my workgroup.” The Cronbach coefficient was 0.86. Furthermore, the result of CFA indicated that the group-based pride scale had high structural validity [${\text{χ}}_{\left(2\right)}^{2}$ = 20.18, CFI = 0.97, TLI = 0.90, and SRMR = 0.03].

### Team Performance

Team leaders assessed their team performance with De Jong and Elfring (2010) three-item scale. This scale also has been used in previous research to rate team performance by team leaders and demonstrated good reliability (Santos and Cardon, 2019). The performance standard contains three aspects: the quantity of work, the quality of work, and the overall efficiency. A sample item was “The amount of work the team produces is high.” The Cronbach coefficient was 0.75 in our study.

### Leader Political Skill

Leader political skill was measured using a six-item scale developed by Ahearn et al. (2004). This scale was self-reported by team leaders at Time 2. A sample item was “It is easy for me to develop a good rapport with most people.” The Cronbach coefficient was 0.82 in our study. In addition, the CFA results indicated that the political skill scale had a good structural validity [${\text{χ}}_{\left(9\right)}^{2}$ = 11.5, CFI = 0.98, TLI = 0.97, and SRMR = 0.04].

### Control Variables

In our data analyses, we controlled several demographic variables. Firstly, we controlled leader demographic information, such as age, gender, and team tenure, given its ties to team performance (Kearney, 2008). Secondly, following prior studies (Owens and Hekman, 2016), we measured and controlled team size, average team gender, and average team age in our analyses. In addition, average team tenure was controlled because past research has shown that average team tenure is related to team performance (Schaubroeck et al., 2007). Importantly, we obtained comparable results both with and without control variables.

### Analytic Procedure

#### Data Aggregation

Because our data analysis was conducted at the team level, the two variables (i.e., identity leadership and group-based pride) that were evaluated by employees need to be aggregated to the team level. To assess the appropriateness of aggregation, we used ANOVA *F*-statistic and calculated the intra-class correlation coefficients (ICCs) and within-group agreement (*r*_wg_) of all individual-level measures (James et al., 1984; Bliese, 2000). ANOVA tests using team as an independent factor suggested that individual ratings on identity leadership and group-based pride differed significantly across teams, with *F* = 2.40, *p* < 0.001 and *F* = 2.93, *p* < 0.001. In addition, ICC (1) and ICC (2), were, respectively, 0.27 and 0.57 for identity leadership, 0.34 and 0.65 for group-based pride. The mean *r*_wg_ was 0.94 and 0.93 for identity leadership and group-based pride. These results in our study suggested identity leadership and group-based pride were appropriate to aggregate to the team level.

#### Analytical Framework

We tested our study hypotheses using two structural equation models (SEMs) in Mplus 8.3 (Muthén and Muthén, 2017). This allowed us to test our hypotheses simultaneously rather than in a piecemeal approach. Specifically, we first conducted a SEM to test the mediation model (Hypotheses 1–3). We then added the moderator (i.e., leader political skill) into the model and tested the moderated mediation model (Hypotheses 4–5). The indirect effect and conditional indirect effect were tested with the Monte Carlo simulation procedure in the software R, which created a 95% bias-corrected CI for each indirect effect (Selig and Preacher, 2008).

## Results

### Confirmatory Factor Analyses

To examine the discriminative validity of our measured variables, we conducted multilevel confirmatory factor analyses (MCFA) in Mplus 8.3. As shown in Table 1, the hypothesized four-factor model yields a good fit to our data, χ^2^ = 99.94, *df* = 45, *p* < 0.001, CFI = 0.95, TLI = 0.92, SRMR = 0.03, and RMSEA = 0.07. This model also was significantly better than a three-factor model combining leader political skill and team performance (Δχ^2^ = 39.10, Δ*df* = 1, *p* < 0.001), a three-factor model combining identity leadership and group-based pride (Δχ^2^ = 154.65, Δ*df* = 1, *p* < 0.001), and a two-factor model combining variables reported by employees and supervisors, respectively (Δχ^2^ = 189.51, Δ*df* = 2, *p* < 0.001). In sum, these results suggested that participants could clearly distinguish the four measures that are used in our study.

**Table 1:** The results of model comparison for a confirmatory factor analysis (CFA).

**Models**	***χ*** **^2^**	***df***	**Δχ^2^(Δ*df*)**	**CFI**	**TLI**	**SRMR**	**RMSEA**
Four-factor model	99.94	45		0.95	0.92	0.03	0.07
Three-factor model (1)	139.04	46	39.10 (1)^***^	0.91	0.87	0.03	0.08
Three-factor model (2)	254.59	46	154.65 (1)^***^	0.79	0.71	0.05	0.13
Two-factor model	289.45	47	189.51 (2)^***^	0.76	0.67	0.05	0.13

*N = 292, four-factor model: all study variables served as independent factors; three-factor model (1): combining leader political skill and team performance to one factor; three-factor model (2): combining identity leadership and group-based pride to one factor; and two-factor model: combining the variables reported by superiors to one factor and combining the variables reported by employees to one factor*.

*** *p < 0.001*.

### Descriptive Statistics

Table 2 shows the means, SDs, and correlations of our research variables at the team level. Coefficient alphas for the overall sample were also presented. The zero-order correlations showed that identity leadership was positively associated to group-based pride (*r* = 0.58, *p* < 0.001) and team performance (*r* = 0.42, *p* < 0.001). Moreover, group-based pride was positively related to team performance (*r* = 0.45, *p* < 0.001).

**Table 2:** Descriptive statistics and correlations.

**Variables**	***M***	***SD***	**1**	**2**	**3**	**4**	**5**	**6**	**7**	**8**	**9**	**10**	**11**
1 Leader age	38.04	5.97											
2 Leader gender	0.63	0.49	0.31^**^										
3 Leader team tenure	5.65	3.65	0.51^***^	0.13									
4 Team average age	28.04	2.54	0.29^**^	0.04	0.25^*^								
5 Team average gender	0.56	0.33	−0.18	−0.05	−0.11	−0.20							
6 Team average tenure	2.17	1.09	0.20	0.03	0.59^***^	0.50^***^	−0.15						
7 Team size	3.59	1.23	0.09	−0.07	0.05	0.11	0.13	0.01					
8 Leader political skill	4.84	0.57	0.27^*^	0.06	0.18	0.17	−0.01	0.05	0.23^*^	**(0.82)**			
9 Identity leadership	4.91	0.41	−0.19	−0.20	−0.05	0.13	0.23^*^	0.13	0.23^*^	0.12	**(0.78)**		
10 Group-based pride	4.54	0.52	−0.09	−0.11	0.20	−0.03	0.14	0.26^*^	0.24^*^	0.05	0.58^***^	**(0.86)**	
11 Team performance	4.69	0.58	0.02	0.004	−0.001	0.31^**^	−0.03	0.24^*^	0.02	0.29^**^	0.42^***^	0.45^***^	**(0.75)**

*N = 81 teams, gender: 0, male; 1, female*.

* *p < 0.05*,

** *p < 0.01*,

*** *p < 0.001*.

*Cronbach's alphas are reported in the parentheses on the diagonal*.

### Test of Mediation

We used three steps to test the hypothesized mediation effect (Preacher and Hayes, 2008). Firstly, the identity leadership significantly predicted group-based pride. Secondly, group-based pride significantly predicted team performance after controlling for the effect of identity leadership. Finally, the indirect effect was significant in the Monte Carlo simulation procedure. As shown in Table 3, after controlling leader age, leader gender, leader's team tenure, team average age, team average gender, team average tenure, and team size, identity leadership is significantly related to group-based pride (*b* = 0.68, *p* < 0.001), thus supporting Hypothesis 1. In support of Hypothesis 2, the positive relationship between group-based pride and team performance was also significant (*b* = 0.49, *p* < 0.01). Moreover, we tested the significance of the mediation model using the Monte Carlo simulation procedure, which showed that the indirect effect was significant with 95% bias-corrected CIs excluding 0 (indirect effect = 0.34, 95% bias-corrected CI = [0.14, 0.58]). Thus, these results supported Hypothesis 3.

**Table 3:** Results of path analysis for mediation.

**Variables**	**Group-based pride**		**Team performance**	
	***B***	***SE***	***B***	***SE***
Leader age	−0.01	0.01	0.01	0.01
Leader gender	−0.01	0.09	0.07	0.11
Leader team tenure	0.03	0.02	−0.04	0.02
Team average age	−0.05^*^	0.02	0.07^**^	0.03
Team average gender	0.06	0.14	−0.08	0.17
Team average tenure	0.11^*^	0.06	0.04	0.07
Team size	0.05	0.04	−0.07	0.04
Identity leadership	0.68^***^	0.11	0.25	0.17
Group-based pride			0.49^***^	0.14

*N = 81 teams. Unstandardized regression coefficients are reported*.

* *p < 0.05*,

** *p < 0.01*,

*** *p < 0.001*.

### Test of Moderated Mediation

**Figure 2:**
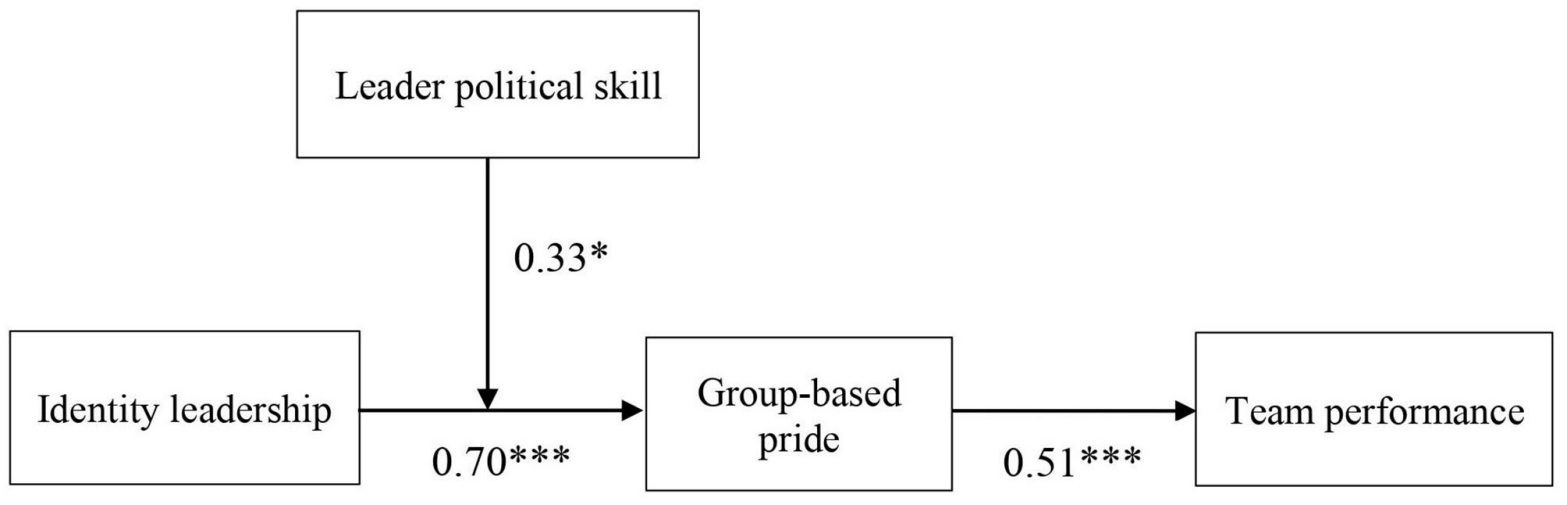
Unstandardized path coefficients of the moderated mediation model. *p < 0.05, ***p < 0.001.

**Figure 3:**
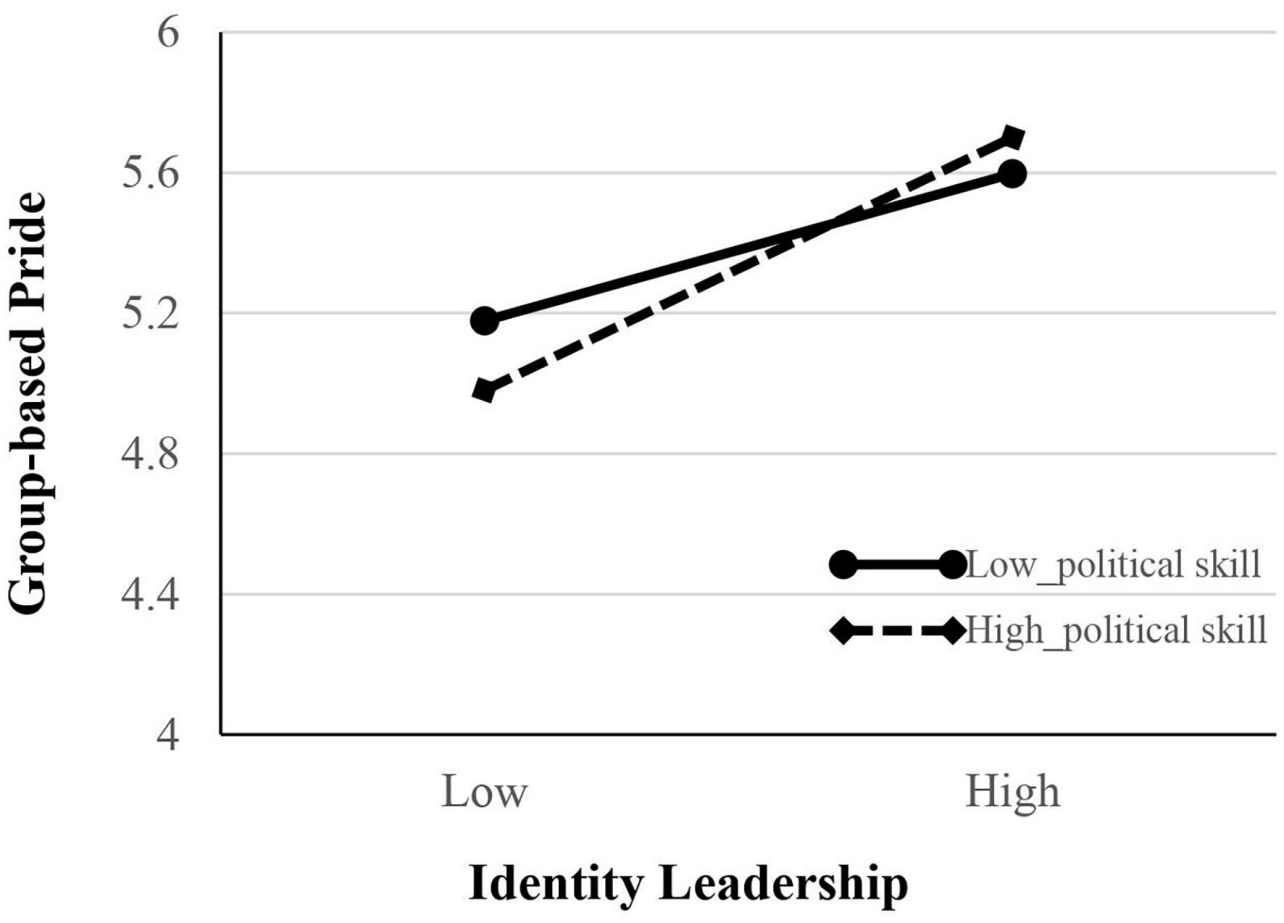
Interaction effect between identity leadership and leader political skill on group-based pride.

As shown in Figure 2, we conducted another SEM to test a moderating effect of leader political skill (Hypothesis 4) and the corresponding moderated mediation effect (Hypothesis 5). Table 4 shows that an interaction term is positive and significant (*b* = 0.33, *p* < 0.05). To better understand the interaction patterns, we depicted interaction plots at one SD above and below the moderator means following Aiken et al. (1991). As shown in Figure 3, the positive relationship between identity leadership and group-based pride is stronger when leader political skill is high (simple slope = 0.88, *t* = 5.97, and *p* < 0.001) rather than low (simple slope = 0.51, *t* = 3.62, and *p* < 0.001). Also, the difference between low and high leader political skills was significant (*t* = 1.99 and *p* < 0.05). To support Hypothesis 5, we utilized the Monte Carlo approach with 95% bias-corrected CIs to test the significance of the moderated mediation model. The results suggested that a conditional indirect effect was significant (index = 0.16, 95% bias-corrected CI = [0.02, 0.41], not contain 0). Specifically, the indirect effect was stronger for those leaders who have high political skill (indirect effect = 0.45, *t* = 3.20, and *p* < 0.01) than those with low political skill (indirect effect = 0.26, *t* = 2.62, and *p* < 0.01). Moreover, the difference of a conditional indirect effect across high and low levels of leader political skill was statistically significant (Δindirect effect = 0.19, 95% bias-corrected CI = [0.02, 0.46]).

**Table 4:** Results of path analysis for moderated mediation.

**Variables**	**Group-based pride**		**Team performance**	
	***B***	***SE***	***B***	***SE***
Leader age	−0.002	0.01	0.01	0.01
Leader gender	0.01	0.09	0.08	0.11
Leader's team tenure	0.03	0.02	−0.05^*^	0.02
Team average age	−0.05^*^	0.02	0.06^*^	0.03
Team average gender	0.10	0.14	−0.07	0.16
Team average tenure	0.10	0.05	0.05	0.07
Team size	0.05	0.04	−0.10^*^	0.04
Identity leadership	0.70^***^	0.11	0.20	0.16
Leader political skill	−0.04	0.08	0.29^**^	0.09
Identity leadership * Political skill	0.33^*^	0.16	0.15	0.20
Group-based pride			0.51^***^	0.13

*N = 81 teams. Unstandardized regression coefficients are reported*.

* *p < 0.05*,

** *p < 0.01*,

*** *p < 0.001*.

## Discussion

Although previous studies have noted the benefits of identity leadership, we know a little about the underlying mechanisms and boundary conditions. Drawing on the social identity approach, the current study suggests that group-based pride plays a key mediating role in the influence of identity leadership on team performance. Identity leadership promotes team members to internalize their group membership and make positive social comparisons between ingroup and outgroup, which elicits group-based pride. Afterwards, group-based pride provides necessary motivation and promotes group cooperation for team performance. Furthermore, this finding is noteworthy that the positive link between identity leadership and group-based pride is stronger when the leader political skill is higher. Politically skilled leaders can access various information and effectively deliver influence on followers so that identity leadership has a strong effect on the pride of followers in the team. Accordingly, we find that leader political skill strengthens the indirect effect of group-based pride by which identity leadership enhances team performance. Thus, leader political skill promotes the effectiveness of identity leadership in affecting team outcomes.

### Theoretical Implications

Utilizing the social identity approach, we seek to examine how identity leadership affects team performance through a mediating role of group-based pride. Furthermore, we find a moderating effect of leader political skill on these associations. These findings provide several theoretical contributions to extant literatures. Firstly, our research contributes to the social identity approach of the leadership literature. Prior research has focused on the influence of leader identity prototypicality on employees and group outcomes or viewing leader identity prototypicality as a boundary condition that moderates the effectiveness of leadership behaviors (e.g., van Knippenberg, 2011; Steffens et al., 2021), ignoring the effect of identity leadership as a higher-order construct. As noted by van Dick et al. (2018), a full scale with four dimensions allows researchers to better capture the richness of identity leadership. Our study fills the gap by documenting the influence of identity leadership on team performance at workplace settings.

Secondly, the current study expands previous work by clarifying how identity leadership contributes to team performance. Although existing research has suggested the influence of the process of leader group prototypicality, we know almost nothing about the underlying mechanisms of global identity leadership achieving effectiveness (Steffens et al., 2014). This investigation draws upon the social identity approach to shed light on the processes by which identity leadership motivates followers to achieve team goals. Thus, our results make important contributions to the theoretical approach by integrating the intergroup emotion theory into the social identity approach. Specifically, our results suggest that group-based pride is an important underlying mechanism through which identity leadership significantly affects team performance.

Finally, we identify leader political skill as a significant boundary condition of leadership affecting group-based pride and team performance. Leaders not only need to “make things happen,” but also utilize personal political skills to “get things done.” Our findings contribute to clarifying the interactive effect of identity leadership and leader political skill in predicting team performance.

### Practical Implications

The results of this study provide several practical implications for managers. Firstly, our results suggest that identity leadership can contribute to team performance. Thus, we recommend that managers should engage in more identity leadership behaviors to obtain better work outcomes. Moreover, identity leadership needs boundary conditions to produce more positive team performance. The current study suggests that leader political skill is a key moderator in the impact of identity leadership on team effectiveness. Politically skilled leaders can access to various information and deliver to their ideas to followers effectively, which strengthens the positive effect of identity leadership. Therefore, managers can improve their political skills to enhance their influence on group members.

Secondly, our results also find that group-based pride drives employees to invest more effort to achieve group goals. We call for future intervention studies to consider these findings and focus on cultivating a sense of pride within the group. Our findings show that identity leadership may provide a feasible pathway to form a sense of shared identity, which provides the basic condition for group-based pride. Researchers and practitioners could use early interventions to improve identity leadership skills (Haslam et al., 2017; Slater and Barker, 2019). Additionally, managers can also use other strategies to cultivate a sense of pride within a group to improve team effectiveness.

## Limitations and Future Directions

As compared with any study, this study has some limitations that should be noted now. Firstly, the possibility for common method bias in self-reported measures should be viewed with appropriate caution, which may inflate correlations and limit causal inferences (Podsakoff et al., 2003). Although this study has used data from different sources, including supervisors and team members, the cross-sectional design prevents us from making causal inferences. In addition, leader political skill is measured at Time 2, whereas group-based pride and team performance are measured at Time 1. Thus, we cannot infer the causal effects of leader political skill on group-based pride and team performance. Although political skill is a relatively stable ability rooted in personal characteristics or personality traits, political skill also can be shaped or developed through training or developmental experience (Ferris et al., 2005; Liu et al., 2007; Munyon et al., 2015). Future research can be benefitted from a more optimized research design. To truly assess the causal direction among identity leadership, leader political skill, group-based pride, and team performance, longitudinal and experimental designs are needed for future research. Secondly, because the job category of our sample is consistent, the generalizability of our results may be a constraint. Despite our sample only consisting of staff in the legal industry, the results based on 81 teams from several companies enable us to believe the robustness of our findings. Considering various work contexts of different industries, future research can examine the generalizability of our findings to other types of companies or other cultures. Thirdly, given that this study only focuses on the mediating effect of group-based pride between identity leadership and team performance, more mechanisms by which identity leadership works should be explored. For instance, group identification, as a cognitive aspect of social identity, may play an important role in the link between identity leadership and team performance. As suggested by van Knippenberg (2011), the influence of leader group prototypicality is tied to group identification. It is reasonable to assume that group identification may mediate or moderate the relationship between identity leadership and team effectiveness. Moreover, given that group-based pride and group identification are both derived from group membership, there may exist a bidirectional or more complex relationship between them. We, therefore, call for future research to examine the role of group identification among these associations. Finally, this study uses a short four-item scale to measure identity leadership, which limits our ability to compare the four dimensions of identity leadership that affect group-based pride and team performance. Additionally, we are not sure whether leader political skill has different moderating effects on the four dimensions of identity leadership. Future research can use the full Identity Leadership Inventory encompasses 15 items to examine the differences in terms of how the four dimensions of identity leadership affect group process and effectiveness.

## Data Availability Statement

The datasets generated for this study are available on request to the corresponding author.

## Ethics Statement

The studies involving human participants were reviewed and approved by Ethics Committee of Renmin University of China. The participants provided oral informed consent to participate in this study.

## Author Contributions

LS proposed the research and conducted project management. GZ and LH organized the data collection. LH carried out the data analysis and wrote the manuscript. BL provided constructive suggestions for the revision of manuscript. All authors contributed to manuscript revision, read, and approved the submitted version.

## Funding

This research was supported by the National Natural Science Foundation of China (Grant No. 71772176).

## Conflict of Interest

The authors declare that the research was conducted in the absence of any commercial or financial relationships that could be construed as a potential conflict of interest.

## Publisher's Note

All claims expressed in this article are solely those of the authors and do not necessarily represent those of their affiliated organizations, or those of the publisher, the editors and the reviewers. Any product that may be evaluated in this article, or claim that may be made by its manufacturer, is not guaranteed or endorsed by the publisher.
